
JOURNAL INFORMATION
==============================
NLM Title Abbreviation: ScientificWorldJournal
Journal ID: ScientificWorldJournal
NLM Journal ID: 101131163
Journal ID: TSWJ

The Scientific World Journal

ISSN: 2356-6140
EISSN: 1537-744X
Publisher: Wiley

ARTICLE INFORMATION
==============================
PMCID: PMC6084218
PMID: 30147610
DOI: 10.1100/tsw.2001.232
Article ID: 582469
Article version: 1
Subjects: Short Report

DNA Methyltransferase I (DNMT1) Inhibition Induces a p21 Dependent Cell Cycle Arrest

Young Juan I. *
Smith James R.
Huffington Center on Aging, Baylor College of Medicine, One Baylor Plaza, Houston, Texas, 77030, USA
*Juan I. Young: jyoung@bcm.tmc.edu
Publication date: 2001
Electronic publication date: 2001 Oct 5
Volume: 1
First page: 131
Last page: 131
Copyright: Copyright © 2001 Juan I. Young and James R. Smith.
Copyright year: 2001
License: This is an open access article distributed under the Creative Commons Attribution License, which permits unrestricted use, distribution, and reproduction in any medium, provided the original work is properly cited.
License URL: https://creativecommons.org/licenses/by/3.0/

==============================
Miami *Nature Biotechnology* Short Reports
*TheScientificWorld* (2001) 1 (S3), 131SR
ISSN 1532-2246; DOI 10.1100/tsw.2001.232

### DNA METHYLTRANSFERASE I (DNMT1) INHIBITION INDUCES A p21 DEPENDENT CELL CYCLE ARREST

Juan I. Young and James R. Smith

Huffington Center on Aging, Baylor College of Medicine, One Baylor Plaza, Houston, Texas, 77030
Jyoung@bcm.tmc.edu

**INTRODUCTION**. Replicative senescence is the terminal non-proliferative state acquired by normal cells in culture after a finite and constant number of divisions. Initially documented in cultures of human fibroblasts, this phenomenon of limited proliferative capacity has been extended to many cell types and has become a model for aging at the cellular level.

Cumulative telomere shortening during cell division has been proposed to be responsible for the induction of cell cycle arrest (1). However, the nature of this anti-proliferative signal and the activation mechanism for the cascades of events ultimately leading to cellular senescence remain unknown.

The cyclin dependent kinase (Cdk) inhibitor p21 plays an essential role in the establishment of the senescent state. It inactivates most of the Cdk-cyclin complexes producing an accumulation of hypophosphorylated Rb no longer able to repress E2f responsive genes. Cloned as a senescent gene, p21 expression increases during in vitro aging. Underscoring its role in senescence, disruption of the p21 gene enables normal human fibroblasts to bypass senescence (2).

Maintenance of methylation patterns in the mammalian genome by DNA (cytosine-5) methyltransferases (DNMT1) is required for normal cell and tissue function. The extent of CpG methylation decreases during serial passage of normal cells in culture (3). Inhibition of DNMT1 in cultured cells induces the expression of p21, a cyclin-dependent kinase (Cdk) inhibitor critical for cells to enter replicative senescence. We investigated the effects of DNMT1 inhibition in normal human fibroblasts and found that it induces an irreversible growth arrest.

**METHODS**. To induce a premature decrease in global DNA methylation levels we treated normal human fibroblasts with 5-aza-2-deoxycytidine (CdR), a potent DNMT1inhibitor, or with DNMT1antisense oligonucleotides (4). We characterized the DNMT1 inhibition induced arrest by analyzing cell cycle distribution, mRNA and protein expression patterns and by testing the response to the introduction of viral oncogenes.

The requirement for p21 on the cell cycle arrest was examined using normal human fibroblasts that lack a functional p21 gene (2).

DNAmethylation levels were measured by HPLC (5). DNA methyltransferase activity was analyzed using hemimethylated substrates as described (6).

**RESULTS.** Cells arrested by DNMT1 inhibition became enlarged and had a flat morphology, exhibited an increased expression of collagenase and p21, and the DNA synthesis block could be overcome by the introduction of the SV40 large T antigen, all characteristics of senescent cells. In contrast, normal human fibroblasts lacking a functional p21 gene fail to undergo cell cycle arrest following DNMT1 inhibition, indicating that p21 is an essential component of this arrest. Furthermore, DNMT1 activity was reduced as cells approached the end of their proliferative potential. These data suggest that DNMT1 could be an integral part of the mechanisms by which cells count the number of cell divisions completed and initiate a signaling cascade that ultimately results in the senescent phenotype.

**REFERENCES.**

1. Harley, C.B., Futcher, A.B., and Greider, C.W. (1990) Nature 345, 458-460
2. Brown, J.P., Wei, W., and Sedivy, J.M. (1997) Science 277, 831-834
3. Wilson, V.L. and Jones, P.A. (1983) Science 220, 1055-1057
4. Fournel, M., Sapieha, P., Beaulieu, N., Besterman, J.M., and MacLeod, A.R. (1999) J. Biol. Chem. 274, 24250-24256
5. Pradhan, S., Bacolla, A., Wells, R.D., and Roberts, R.J. (1999) J. Biol. Chem. 274, 33002-33010
6. Adams, R.L., McKay, E.L., Craig, L.M., and Burdon, R.H. (1979) Biochem. Biophys. Acta 561, 345-357
